# *Nourish*: A pilot program to support self-Efficacy, learning, and wellness during USMLE step 1 preparation

**DOI:** 10.1080/10872981.2022.2153781

**Published:** 2022-12-10

**Authors:** Nishad C. Sathe, Patricia A. Carney, Megan Furnari

**Affiliations:** a Department of Dermatology and Medicine, University of Minnesota Medical School, Minneapolis, MN, USA; b Department of Family Medicine, Oregon Health & Science University, Portland, OR, USA; c Department of Pediatrics, Oregon Health & Science University, Portland, OR, USA

**Keywords:** Undergraduate medical education, medical student wellness, USA medical licensing exam, mentorship

## Abstract

**Purpose:**

Medical trainees experience significant exam-related stress, such as preparing for the USA Licensing Medical Examination Step 1, which often negatively affects emotional health. *Nourish*, a novel Step 1 support program, was designed to foster improved self-efficacy and well-being during the process of studying for and taking the exam. *Nourish* was piloted at Oregon Health & Science University between December 2018 and February 2019.

**Methods:**

Program elements were guided by Self-Efficacy Theory and included community building, wellness support, peer tutoring and social persuasion. Program evaluation included pre- and post-program surveys. Participation was optional and included 46 of 154 students (30%) with 40 of the 46 students (87%) completing pre and post evaluations. The pre-survey was given during the *Nourish* orientation in December prior to the Step 1 study period, and the post-survey was given in early February when most students had taken their exam but none had received their scores.

**Results:**

While summary self-efficacy scores increased between baseline and post program (24.9 vs 27.7, p < 0.001), summary emotional health scores worsened (8.15 vs 8.75, p = 0.03). Summary scores for physical health also dropped but this difference was not statistically significant. Summary perceived stress scores increased from 15.5 at baseline to 23.7 post program (p < 0.001). All students who routinely participated in *Nourish* passed their USMLE Step 1 exam. One student who participated only in the orientation session did not pass.

**Conclusion:**

*Nourish* appeared to improve self-efficacy, even though students reported being stressed with low emotional health. The program appeared to help students align task demands with their own personal resources and set reasonable expectations and strategies to pass the exam. Medical schools should consider similar peer- and faculty mentor-based wellness and tutoring programs to support medical students while they work to achieve academic success.

## Introduction

Preparing for the U.S. Medical Licensing Exam (USMLE) is extremely stressful for medical students [1–4]. This is due, in part, to the fact that both subjective well-being and academic achievement play a major role in learners’ lives [5]. For medical students, their decisions about their future discipline and likelihood of matching to their top choice can be greatly affected by their scores on USMLE Steps 1 and 2. Stressful experiences have persisted even after Step 1 became pass/fail [6]. Many medical schools now include a four to eight week preparation period after the end of the foundational courses in medicine and before clerkship rotations begin to allow students to ready themselves for Step 1 [7]. Traditionally, these preparation periods included dedicated study time either independently or in small groups, along with practice test banks and other resources [7,8].

Although medical student wellness in general has received significant attention over the past several years [9–12], very little research has focused on student well-being during the Step 1 preparation period. Four notable studies were recently published. Tackett et al [13, 2022] surveyed second year medical students at four U.S. schools after each schools’ preparation period for Step 1 and concluded that many student preparation periods are characterized by personal and social deprivation that may be worsened by several stressors including financial costs of preparation materials. These authors called on reforming these programs to better address student well-being. Lynch et al [14, 2022] developed and implemented a student near-peer mentorship program designed to offer emotional support and wellness advice while not providing academic counseling. They reported reducing medical student burnout while fostering a supportive community during a typically isolating and emotionally challenging time [14]. Schwartz et al [15, 2018] and Gonzalez et al [16, 2019] both describe near-peer programs offering academic support only through content review sessions and practice questions.

**Figure 1.**
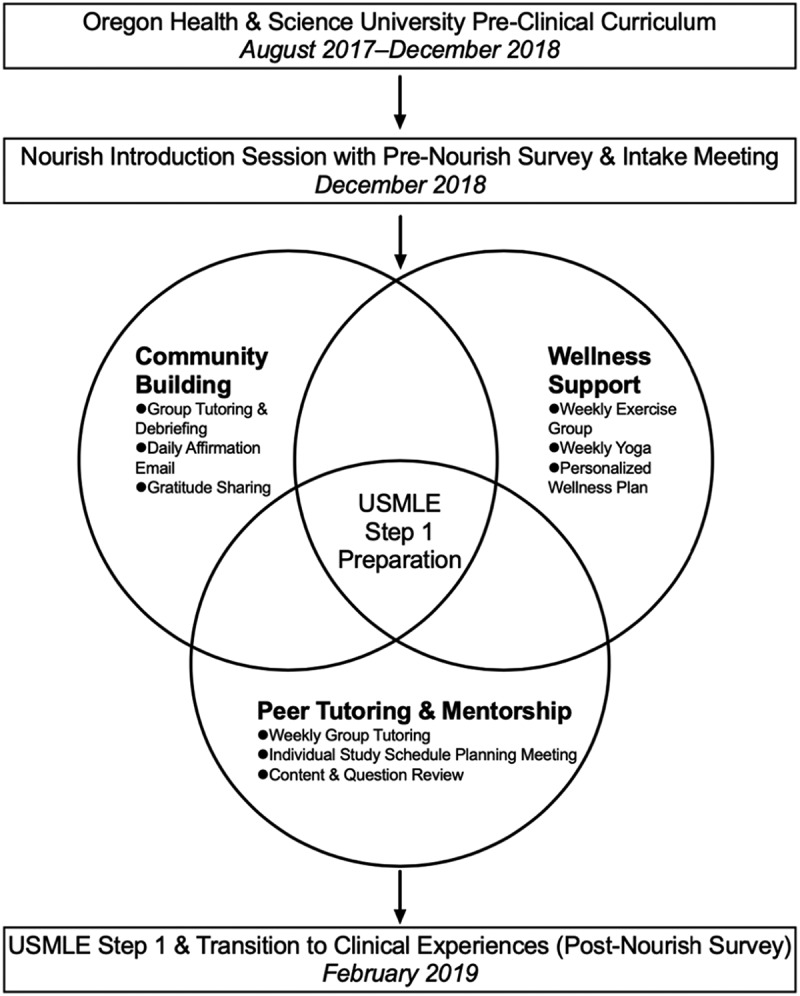
*Nourish* program overview. Timeline for second-year medical students’ (Class of 2021) participation in *Nourish.*

Conceptually, well-being is a complex higher order multifactorial construct with many interactive domains [17]. Additionally, populations and settings, such as universities or medical schools and different types of learners, are key features likely to affect different aspects of well-being [18]. Interventions and their related measures to improve well-being should be theory driven. We developed and piloted *Nourish*, a novel USMLE Step 1 preparation program designed to provide *both* academic and wellness support to medical students during the designated study period (Figure 1). Self-Efficacy Theory guided this work where medical students’ beliefs in their capabilities to exercise control over their own functioning and over events in their lives provides a foundation for motivation, well-being, and personal accomplishment [19].

## Methods

This mixed-methods prospective cohort pilot study was designed to explore the impact that *Nourish* had on participants’ self-efficacy, perceived stress, and physical and emotional well-being, during USMLE Step 1 preparation. These measures were chosen based on our intervention components and because prior research has shown that high self-efficacy is associated with several daily life benefits, including resilience to adversity and stress, healthy lifestyle habits, improved work performance, and educational achievement [20–22]. All study activities were reviewed and approved by the OHSU Institutional Review Board (IRB# 19,244).

Learning Setting and Program Design

**Figure 2.**
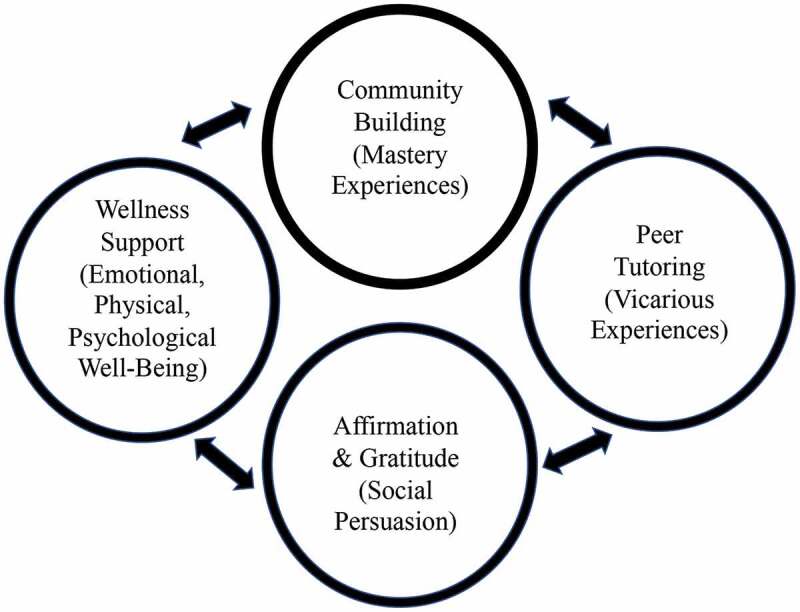
Nourish program framework based upon the self-Efficacy Theory.

Oregon Health & Science University (OHSU), the only MD-granting medical school in Oregon, enrolls approximately 155 students per year into its time-varying competency-based curriculum, which has been in place since 2014. As mentioned, Self-Efficacy Theory provided the intervention and measurement framework where four main sources of influence build self- efficacy: 1) Mastery experiences or students learning that they are capable of acquiring new skills; 2) Vicarious experiences or students experiencing positive peer role models who successfully achieved a task; 3) Social persuasion or receiving positive feedback while undertaking a complex task; and 4) Emotional, physical and psychological well-being which can influence how students feel about their abilities [23]. Thus, the program had four features: 1) community building for Mastery Experiences, 2) peer tutoring for Vicarious Experiences; 3) daily affirmation emails and gratitude sharing for Social Persuasion; and 4) wellness support for Emotional, physical and psychological well-being, (Figure 2). This educational model was supported by students, faculty, and institutional leadership.

The *Nourish* team included a third-year medical student, a first-year medical student who was a certified yoga teacher, and a faculty member who serves as Director of Wellness at the medical school. *Nourish was provided to participants at no cost*. The Director of Wellness devoted 0.20 FTE to oversee *Nourish* during the five weeks it ran. A $10,000 external grant was used to pay the third-year student $20 per hour for 200 hours of tutoring, and the first-year student yoga instructor was paid $75 per class for 5 yoga classes.

*Nourish* was an optional program available to all 154 second-year medical students during their USMLE Step 1 study period. The structured *Nourish* program ran from January to early February 2019. Enrollment occurred at the December *Nourish* orientation session. Enrollment was expected to include students who perceived they may be at risk of not passing USMLE Step 1 on their first attempt based on their OHSU mock exam scores. The mock exam is created by OHSU faculty and consists of selected National Board of Medical Examiners (NBME) questions to help simulate the Step 1 experience and give students a general idea of their likely performance.

During *Nourish*, a weekly email went out with the dates and times of optional activities. In-person activities were held at the medical school. Group tutoring occurred weekly for 2-hours on historically challenging topics for OHSU students, based on cumulative generic cohort performance reports. The focus was learning high-yield content and practicing NBME-style questions in that content area. Individual tutoring sessions focused on study skills and knowledge development with an intentionally selected peer tutor to increase student engagement and the known similar efficacy outcomes compared to faculty tutors [24].

Personal wellness sessions focused on stress reduction techniques, mind-body exercises, sleep hygiene, breathing exercises, healthy study routines, and cultivating a growth mindset. Yoga occurred once per week. Daily affirmation e-mails were sent to students by the Director of Wellness and included inspiring quotes and mindfulness activities with an emphasis on self-efficacy [25]. Gratitude sharing occurred via a Google document every Friday to enhance positive emotions [26].

Survey Instrument Development

Participant assessments measured learner self-efficacy, perceived stress, physical and emotional health, and performance on USMLE Step 1. Validated measures selected for this pilot contained a total of 27 variables included on a pre- and post-program survey. Nine items assessed learner self-efficacy and were adapted from the validated Generalized Self-Efficacy Scale, with reported Cronbach’s alpha coefficients between 0.76 and 0.90 [27]. We assessed Cronbach’s alpha for our adapted scale, which revealed coefficients of 0.83 for the pretest and 0.86 for the post test. We used ten items from the Perceived Stress Scale (PSS) (Cronbach’s alpha of 0.84–0.86) [28,29], and eight items from the validated the Short-Form 8 (SF-8) to assess physical and emotional health (Cronbach’s alpha of between 0.79 and 0.85). The SF-8 and PSS are both well-validated for use with medical students [30,31]. Analyses of data from all validated scales followed the scoring procedures published by authors who developed and validated the items, including items that were reversed scored. The post-program survey additionally included open-ended questions about students’ experiences with the program, along with their level of engagement. Both pre- and post-program surveys are provided in **Appendix 1**. The pre-program survey provided baseline data. We did not include a control group in this pilot program, as we felt it unethical to withhold supportive interventions for students at high risk of failing Step 1.

Surveys and test scores were collected and deidentified by an OHSU testing coordinator who replaced students’ names with a study identifier for linkage in paired analyses. Mock USMLE Step 1 scores and USMLE Step 1 scores were linked to the *Nourish* program participants (40 who completed both surveys) and non-participants (the rest of the medical school class or 114 students). The pre-program survey was given just prior to the start of the designated study period in December 2018. The post-program survey was given in early February 2019, when most students had just completed their Step 1 exam. It is important to note that 32 students of the 154 students in the entire class (20.7%) delayed taking their exam in 2019, a decrease from 2018 when 38 students delayed their exams. This means that the post-program survey was administered while some participants were still studying for their exam. We are unable to determine the exact numbers of *Nourish* students who delayed taking the exam, as this information is confidential. We do know that no one knew their exam scores when they took the post-program survey in early February. Conversations about delaying the exam and prolonging the study period beyond the *Nourish* program were referred to OHSU Assistant Deans for Student Affairs. Four questions from the qualitative survey were asked of participants: 1) ‘Should Nourish be offered again for future classes?’, 2) ‘What was most valuable about Nourish to you?’, 3) ‘What will you take with you and continue to use from Nourish?’ and 4) ‘What would you change for next year?’.Quantitative and Qualitative Analysis:

Descriptive statistics, including means, standard deviations, and ranges were calculated for each survey item. Four Perceived Stress Scale items were reverse scored according to scoring instructions for this instrument [30]. Summary scores were calculated for each domain measured. In addition, comparisons for pre-and post-program data for learner self-efficacy, perceived stress, and physical and emotional health, were run using paired t-tests. All tests were two-tailed and alpha levels were set at 0.05 for determining statistical significance. Quantitative analyses were conducted using SPSS Version 27 (IBM Corp., Chicago). We logged the number of students in attendance at the *Nourish* offerings and the number of individual interactions that occurred between students and the third-year tutor or Director of Wellness during the *Nourish* program to understand engagement. These interactions included emails, text messages, phone calls and in-person meetings. The number of interactions and attendance beyond attending the orientation session was not linked to the survey data or Step 1 scores.

Qualitative analyses were conducted using classical content analysis to identify emergent themes in responses to the open-ended questions [32].

## Results

Forty-six students enrolled in *Nourish* and, of these, 40 (87%) completed the pre- post program assessments. Thirty-three percent of the 40 participants were male with average age of 26.9 years (S.D. = 2.81 years), and 67% were female with average age 26.2 years (S.D. = 2.3). No statistical difference was found for age according to gender (p = 0.43) (*Data not shown*).

An average of 7.5 interactions (range 0–35) occurred with the *Nourish* team per participant during the study period. The most popular form was e-mail between the Director of Wellness and participants and text messaging or in-person meetings for the third-year medical student tutor. Weekly group tutoring attendance averaged 8 students (17% of the total participants), with a range of 3 to 17 students depending on the week and topic. Weekly yoga was attended by an average of 4 students (range 3–5). Virtual gratitude sharing had variable engagement, with most comments occurring during the first few weeks, then a steady decline, and the offering was stopped by week four. Most students rated their own engagement in the program as 3–5 on the post-survey (scale of 1 to 10, with 1 attending the orientation and 10 attending at least every type of event once).

Summary scores for learner self-efficacy statistically improved over time, with a mean of 24.9 at baseline and 27.7 post program (p < 0.001) (Table 1). Summary physical health scores did not change over time (Table 2), though summary emotional health score worsened between baseline and post program (mean of 8.15 at baseline and 8.75 post program (p = 0.03)) (Table 2). Summary mean perceived stress scores increased significantly from 15.5 at baseline to 23.7 post program (p < 0.001) (Table 3).

**Table 1.** Learner self-Efficacy scores at baseline and post program.

Learner Self-Efficacy Variables ^a^	Baseline Mean (SD†) (n = 40)	Post Program Mean (SD) (n = 40)	p value
I can handle whatever Step 1 question comes my way.	2.10 (0.84)	2.78 (0.58)	<0.001
If information is unknown to me, I can find the means and ways to get the information I need.	3.34 (0.61)	3.30 (0.56)	0.74
I can solve most Step 1 questions if I invest the necessary effort.	2.88 (0.72)	3.05 (0.60)	0.18
Thanks to my resourcefulness, I can learn about topics I have never seen before independently.	3.25 (0.63)	3.43 (0.59)	0.70
When I am confronted with a clinical case, I can find several causative agents.	2.68 (0.57)	3.08 (0.57)	<0.001
If I feel challenged by a clinical case, I can think of a good way to find the answer.	2.68 (0.67)	3.08 (0.76)	<0.001
I am confident that I could deal efficiently with unknown Step 1 question.	2.33 (0.81)	2.92 (0.58)	<0.001
I can remain calm when facing clinical vignettes because I can rely on my abilities.	2.73 (0.75)	2.13 (0.65)	0.002
I am certain that I can accomplish my Step 1 learning goals.	2.90 (0.63)	2.90 (0.63)	1.00
**Summary Learner Self-Efficacy Scores**	**24.9 (4.14)**	**27.7 (3.84)**	**<0.001**

***^a^*** Scale: 1 = Not true; 2 = Hardly true; 3 = moderately true; 4 = Exactly true

†SD = Standard Deviation

**Table 2.** SF-8 responses for emotional and physical health at baseline and post program.

Wellness Variables *Question Stem: During the past 4 weeks,*	Baseline Mean (SD†) (n = 40)	Post Program Mean (SD) (n = 40)	p value
How much did physical health problems limit your usual physical activities (such as walking or climbing stairs)? **^a^**	1.58 (0.81)	1.23 (0.53)	0.03
How much difficulty did you have doing your daily work, both at home and away from home, because of your physical health? **^a^**	1.40 (0.63)	1.25 (0.49)	0.18
How much did your physical or emotional problems limit usual social activities with family or friends? **^a^**	1.90 (0.94)	2.28 (1.03)	0.05
How much bodily pain have you had during the past 4 weeks? **^b^**	2.08 (0.86)	2.00 (0.93)	0.58
***Physical Wellness Summary Score***	6.97 (2.60)	6.74 (2.10)	0.58
How much did personal or emotional problems keep you from doing your usual work, school or other daily activities? **^a^**	2.03 (0.83)	2.33 (0.97)	0.10
How much energy did you have? **^c^**	3.15 (0.70)	3.15 (0.80)	1.00
How much have you been bothered by emotional problems (such as feeling anxious, depressed or irritable)? **^d^**	2.98 (0.92)	3.28 (1.06)	0.04
***Emotional Wellness Summary Score***	8.15 (1.31)	8.75 (1.63)	0.03
***Overall, how would you rate your health ^e^***	3.84 (0.96)	3.70 (1.15)	0.54

†SD = Standard Deviation

**^a^** Scale: 1 = Not at all; 2 = Very Little; 3 = Somewhat; 4 = Quite a Lot; 5 = I Could Not do Physical Activities

**^b^** Scale: 1 = None; 2 = Very Mild; 3 = Mild; 4 = Moderate; 5 = Severe

**^c^** Scale: 1 = A Little; 2 = Some; 3 = Quite a Lot; 4 = Very Much

**^d^** Scale: 1 = Not at All; 2 = Slightly; 3 = Moderately; 4 = Quite a Lot

**^e^** Scale: 1 = Very poor; 2 = Poor; 3 = Fair; 4 = Good; 5 = Very good; 6 = Excellent

**Table 3.** Perceived stress scale responses for emotional and physical health at baseline and post program.

Perceived Stress Variable ^a^ *Question Stem: In the last month,*	Baseline Mean (SD†) (n = 40)	Post Program Mean (SD†) (n = 40)	p value
How often have you been upset because of something that happened unexpectedly?	1.55 (0.71)	2.70 (0.88)	<0.001
How often have you felt that you were unable to control the important things in your life?	1.50 (0.93)	1.65 (1.15)	0.53
How often have you felt nervous and ‘stressed’?	2.85 (0.86)	4.18 (0.78)	<0.001
How often have you felt confident about your ability to handle your personal problems? **^R^**	1.08 (0.69)	1.20 (0.79)	0.68
How often have you felt that things were going your way? **^R^**	1.40 (0.71)	1.05 (1.09)	0.08
How often have you found that you could not cope with all the things that you had to do?	1.40 (0.71)	2.73 (1.11)	<0.001
How often have you been able to control irritations in your life? **^R^**	1.03 (0.73)	3.83 (0.93)	<0.001
How often have you felt that you were on top of things? **^R^**	1.65 (0.74)	1.13 (1.02)	0.002
How often have you been angered because of things that were outside of your control?	1.48 (0.91)	2.35 (0.83)	<0.001
How often have you felt difficulties were piling up so high that you could not overcome them?	1.60 (0.84)	2.90 (1.20)	<0.001
***Summary Perceived Stress Score ^b^***	15.53 (4.88)	23.7 (4.70)	<0.001

†SD = Standard Deviation

**^a^** Scale: 0 = Never; 1 = Almost Never; 2 = Sometimes; 3 = Fairly Often; 4 = Very Often

**^R^** Reverse Scored

**^b^** Summary score interpretation: 0–13 considered low stress, 14–26 considered moderate stress, 27–40 considered high stress

All participants indicated on the open-ended survey questions that *Nourish* should be offered again for future classes. The most valuable elements about the program included feeling part of a community (19/40), decreasing anxiety (14/40), and receiving more individualized guidance (13/40). Participants said they would continue to use the following from the *Nourish* experience: the value of community and not feeling socially isolated in stressful times, being able to ask for help from faculty and peers, perspective regained from affirmation emails, and study skills. Program changes for the next year included significantly more individual tutoring time, starting the program sooner than the designated study period, and providing more physical activity options to better align with schedules and needs.

Mock USMLE Step 1 scores were 61.24 (S.D. = 7.53) for *Nourish* participants and 64.46 (S.D. = 9.48) for non-*Nourish* participants. The range of mock USMLE Step 1 scores for *Nourish* participants was 47–74 and for non-participants was 42–91. The correlation coefficients between the mock USMLE Step 1 and actual USMLE Step 1 performance were not significantly different between *Nourish* participants (R^2^ = 0.55) and non-participants (R^2^ = 0.57). The actual average Step 1 scores were 225.2 (S.D. = 15.7) for *Nourish* participants and 231.1 (S.D. = 16.6) for non-participants, with 1 *Nourish* participant, who only attended the orientation *Nourish* offering, and 1 non-participant’s score falling below the national passing threshold. Average Step 1 scores at OHSU between 2014 to 2020 ranged from 225–230, similar to national averages.

## Discussion

*Nourish* is a novel program in that it supports both wellness and learning experiences for participants and is the first program to our knowledge that provides this scope. Prior programs either focused on only wellness or only peer tutoring [14–16]. Self-Efficacy Theory, the guiding framework for *Nourish*, drove the intervention elements and study measures. Based on post compared to pre-program measures, we noted a statistically significant increase in self-efficacy while also finding a decline in emotional health and increased perceived stress. This finding is inconsistent with other research that has linked improved self-efficacy with both improved wellness, lowered stress and higher academic achievement [20–22]. It may be that the stress associated with studying for and taking Step 1 could not be fully mitigated by improving self-efficacy. It is a high-stakes exam, the timing of which is minimally flexible and thus not easy to control. Another possibility is that some of the students participating in *Nourish* had not yet completed their exam at the time of the post-program survey, which could have been a driver of the worsening emotional health and increased perceived stress scores. In addition, even those who had completed their exams did not have their scores back when the post-program survey was given, thus capturing potential stress from anticipatory anxiety while awaiting the test result. It is unknown what the stress and wellness scores would have been among participants if they had chosen not to participate in *Nourish*, though we suspect they may have been even higher.

Lynch et al. in the ‘Step Siblings’ program focused on peer wellness support only and found that participants did feel less stressed but this program did not include academic support [13]. Participants and peer mentors reported wanting more academic support in the program as they felt that was critical to wellness in the Step 1 study experience [13]. The Schwartz et al. pilot program focused on academic support only from student tutors and saw a 6.57 point increase in Step 1 scores for participants and increased likelihood of passing [15]. Benefits of the program included institutional support and no cost for the Step 1 preparation. One of the challenges in the program was the varying cohort cooperation between the two pilot years, which could have impacted their outcomes [15]. The second cohort had a decreased sense of unity around a common goal and felt less ownership compared to the first cohort who helped to create and launch the program [15]. Adding a wellness component may have improved cohort cohesion and created a greater sense of self-efficacy for participants.

The *Nourish* qualitative data revealed that students felt a strong sense of community and personal support. Students also reported the most valuable program component was decreased anxiety; thus, we speculate that emotional health decline and perceived stress increase in the post-survey may have been even worse without the program. We realize post-program survey timing could be revisited to best capture the impact of *Nourish*. Neither of the programs described by Lynch et al. or Schwartz et al. measured wellness or self-efficacy using validated instruments. Both programs were initiated and run by other medical students, while *Nourish* had the student tutor but relied heavily on the Director of Wellness to coordinate program activities, which is likely a better model for the longevity of the program over time.

A limitation of the pilot is that despite tracking numbers in attendance for activities, we neglected to link student identifiers to survey responses and, thus, don’t know which participants were most engaged in the program. We did ask participants to self-assess engagement in the post-survey, but this is subject to recall bias. Another limitation is the possibility of response bias on the part of those who chose to complete the surveys versus those who did not. An additional limitation includes the small number of students in the program, 46 enrolled and 40 completed pre- and post-surveys. The total class size was 154, so participation was 30%. As a pilot program, *Nourish* was initially designed to support about 50 students. In the future, we plan to increase enrollment and expand the number of tutors to assist them. The final limitation is that we did not include a control group, as we felt it unethical to withhold supportive interventions for students at high risk of failing Step 1.

The primary goal of *Nourish* was to help students feel well prepared for the exam by supporting both learning and wellness, which we hoped would contribute to a higher Step 1 score. Based on the self-efficacy data and qualitative comments, *Nourish* students did report a more supported experience. The *Nourish* framework could be a possible new approach to help students during this critical period of exam preparation. Future directions for the next *Nourish* cohort include having more student tutors, getting more information about each participant’s unique needs in the pre-survey, and starting the program sooner based on qualitative responses. One tutor helping 40 students was not optimal, and desire for more frequent individual tutoring became evident from the volume of student interactions the tutor had and on the post-program survey feedback. The plan for the next cohort is to increase the number of student tutors to allow for a more customized peer learning experience that starts prior to the designated study time. We also lacked relevant background information on individuals’ needs, previous testing experiences, and goals. Gathering this data in the future will help tutors support individual student needs.

In conclusion, the *Nourish* pilot program’s unique focus on both wellness and academic support appears helpful and to increase student’s sense of self-efficacy. More research is needed to fully understand how best to support student learning, mitigate stress, and promote emotional well-being during stress-inducing examination study periods.

## Appendix 1.

**Pre and Post Surveys**

**Nourish Pre-Survey**

**Date of Completion: __ __/ __ __/ __ __**

**Name:**

**Age:**

**Gender:**

**1. How prepared do you feel for the Step 1 study period?**

**2. What do you hope to gain from the Nourish program, your own goals?**

**3. What wellness practices do you use the most in your own medical school life currently?**

**Please answer the following survey items about learning**:

**Table:**

Item	Not at all True	Hardly True	Moderately True	Exactly True
I can handle whatever Step 1 question comes my way.				
If information is unknown to me, I can find the means and ways to get the information I need.				
I can solve most Step 1 questions if I invest the necessary effort.				
Thanks to my resourcefulness, I can learn about topics I have never seen before independently.				
When I am confronted with an clinical case, I can find several causative agents.				
If I feel challenged by a clinical case, I can think of a good way to find the answer.				
I am confident that I could deal efficiently with unknown Step 1 question.				
I can remain calm when facing clinical vignettes because I can rely on my abilities.				
I am certain that I can accomplish my Step 1 learning goals.				

**Instructions**: The following eight questions ask for your views about your health ***during the last four weeks. Please note that the response scales change frequently, so please review your response choices carefully as you complete these questions.***

**Table:**

SF-8	Very Poor	Poor	Fair	Good	Very Good	Excellent
Overall, how would you rate your health during the past 4 weeks?	0	1	2	3	4	5

**Table:**

SF-8	Not at all	Very Little	Somewhat	Quite a lot	Could not do physical activities
During the past 4 weeks, how much did physical health problems limit your usual physical activities (such as walking or climbing stairs)?	0	1	2	3	4
During the past 4 weeks, how much difficulty did you have doing your daily work, both at home and away from home, because of your physical health?	0	1	2	3	4
During the past 4 weeks, how much did your physical health or emotional problems limit usual social activities with family or friends?	0	1	2	3	4
During the past 4 weeks, how much did personal or emotional problems keep you from doing your usual work, school or other daily activities?	0	1	2	3	4

**Table:**

SF-8	None	Very Mild	Mild	Moderate	Severe	Very Severe
How much bodily pain have you had during the past 4 weeks?	0	1	2	3	4	5

**Table:**

SF-8	None	A Little	Some	Quite a Lot	Very Much
During the past 4 weeks, how much energy did you have?	0	1	2	3	4

**Table:**

SF-8	Not at all	Slightly	Moderately	Quite a lot	Extremely
During the past 4 weeks, how much have you been bothered by emotional problems (such as feeling anxious, depressed or irritable)?	0	1	2	3	4

**Instructions**: The following 10 questions ask you about your feelings and thoughts ***during the last month***. In each case, you will be asked to indicate by circling how often you felt or thought a certain way.

**Table:**

PSS	Never	Almost Never	Sometimes	Fairly Often	Very Often
In the last month, how often have you been upset because of something that happened unexpectedly?	0	1	2	3	4
In the last month, how often have you felt that you were unable to control the important things in your life?	0	1	2	3	4
In the last month, how often have you felt nervous and ‘stressed’?	0	1	2	3	4
In the last month, how often have you felt confident about your ability to handle your personal problems?	0	1	2	3	4
In the last month, how often have you felt that things were going your way?	0	1	2	3	4
In the last month, how often have you found that you could not cope with all the things that you had to do?	0	1	2	3	4
In the last month, how often have you been able to control irritations in your life?	0	1	2	3	4
In the last month, how often have you felt that you were on top of things?	0	1	2	3	4
In the last month, how often have you been angered because of things that were outside of your control?	0	1	2	3	4
In the last month, how often have you felt difficulties were piling up so high that you could not overcome them?	0	1	2	3	4

## Appendix 2.

**Nourish Post-Survey**

**Date of Completion: __ __/ __ __/ __ __**

**Name:**

**Age:**

**Gender**:

1. What was the most valuable about Nourish to you, check below?

Feeling of community __

More personal guidance___

Decreased your anxiety ___

More prepared for Step 1___

Fill in your own reply:

2. Rate your level of involvement with the program from 1 to 10 (1- didn’t attend anything after first orientation, 10- did nearly all activities/opportunities at least once)?

3. What Nourish activities did you attend and how often? Circle the activities you would keep?

Personal Email Check-Ins:____

Tutoring: _____

Run:____

Yoga:____

Guided Relaxation:___

Gratitude Google Doc on Fridays:_____

In person guidance with Dr. Furnari/Nishad: ______

3. What will you take with you and continue to use from Nourish?

4. What would you change for next year? Are there elements that we need more of or less of? What did we forget?

5. Should we offer Nourish again to future students?

Yes___ No___

**Please answer the following survey items about learning**:

**Table:**

Item	Not at all True	Hardly True	Moderately True	Exactly True
I can handle whatever Step 1 question comes my way.				
If information is unknown to me, I can find the means and ways to get the information I need.				
I can solve most Step 1 questions if I invest the necessary effort.				
Thanks to my resourcefulness, I can learn about topics I have never seen before independently.				
When I am confronted with an clinical case, I can find several causative agents.				
If I feel challenged by a clinical case, I can think of a good way to find the answer.				
I am confident that I could deal efficiently with unknown Step 1 question.				
I can remain calm when facing clinical vignettes because I can rely on my abilities.				
I am certain that I can accomplish my Step 1 learning goals.				

**Instructions**: The following eight questions ask for your views about your health ***during the last four weeks. Please note that the response scales change frequently, so please review your response choices carefully as you complete these questions.***

**Table:**

SF-8	Very Poor	Poor	Fair	Good	Very Good	Excellent
Overall, how would you rate your health during the past 4 weeks?	0	1	2	3	4	5

**Table:**

SF-8	Not at all	Very Little	Somewhat	Quite a lot	Could not do physical activities
During the past 4 weeks, how much did physical health problems limit your usual physical activities (such as walking or climbing stairs)?	0	1	2	3	4
During the past 4 weeks, how much difficulty did you have doing your daily work, both at home and away from home, because of your physical health?	0	1	2	3	4
During the past 4 weeks, how much did your physical health or emotional problems limit usual social activities with family or friends?	0	1	2	3	4
During the past 4 weeks, how much did personal or emotional problems keep you from doing your usual work, school or other daily activities?	0	1	2	3	4

**Table:**

SF-8	None	Very Mild	Mild	Moderate	Severe	Very Severe
How much bodily pain have you had during the past 4 weeks?	0	1	2	3	4	5

**Table:**

SF-8	None	A Little	Some	Quite a Lot	Very Much
During the past 4 weeks, how much energy did you have?	0	1	2	3	4

**Table:**

SF-8	Not at all	Slightly	Moderately	Quite a lot	Extremely
During the past 4 weeks, how much have you been bothered by emotional problems (such as feeling anxious, depressed or irritable)?	0	1	2	3	4

**Instructions**: The following 10 questions ask you about your feelings and thoughts ***during the last month***. In each case, you will be asked to indicate by circling how often you felt or thought a certain way.

**Table:**

PSS	Never	Almost Never	Sometimes	Fairly Often	Very Often
In the last month, how often have you been upset because of something that happened unexpectedly?	0	1	2	3	4
In the last month, how often have you felt that you were unable to control the important things in your life?	0	1	2	3	4
In the last month, how often have you felt nervous and ‘stressed’?	0	1	2	3	4
In the last month, how often have you felt confident about your ability to handle your personal problems?	0	1	2	3	4
In the last month, how often have you felt that things were going your way?	0	1	2	3	4
In the last month, how often have you found that you could not cope with all the things that you had to do?	0	1	2	3	4
In the last month, how often have you been able to control irritations in your life?	0	1	2	3	4
In the last month, how often have you felt that you were on top of things?	0	1	2	3	4
In the last month, how often have you been angered because of things that were outside of your control?	0	1	2	3	4
In the last month, how often have you felt difficulties were piling up so high that you could not overcome them?	0	1	2	3	4
